# Effective bifidogenic growth factors cyclo-Val-Leu and cyclo-Val-Ile produced by *Bacillus subtilis* C-3102 in the human colonic microbiota model

**DOI:** 10.1038/s41598-020-64374-w

**Published:** 2020-05-05

**Authors:** Misaki Hatanaka, Hiroto Morita, Yumi Aoyagi, Kengo Sasaki, Daisuke Sasaki, Akihiko Kondo, Teppei Nakamura

**Affiliations:** 1 Research & Development Dept, Asahi Calpis Wellness Co., Ltd., 4-1, 2-chome, Ebisu-Minami, Shibuya-ku, Tokyo, 150-0022 Japan; 2 Department of Microbiological Flora Technology, Core Technology Laboratories, Asahi Quality and Innovations Co., Ltd. 5-11-10 Fuchinobe, Chuo-ku, Sagamihara-shi, 252-0206 Japan; 3 Graduate School of Science, Technology and Innovation, Kobe University, 1-1 Rokkodai-cho, Nada-ku, Kobe, Hyogo, 657-8501 Japan

**Keywords:** Bacteria, Microbiome

## Abstract

*Bifidobacterium* species are known to fulfill important functions within the human colon. Thus, stimulating the activity of bifidobacteria is important to maintain host health. We revealed that culture supernatants of *Bacillus subtilis* C-3102 (referred to as C-3102) stimulated the growth of *Bifidobacterium* species. In this study, we isolated and identified six bifidogenic growth factors, which were cyclo (D-Val-D-Ile), cyclo (L-Val-D-Ile), cyclo (D-Val-L-Ile), cyclo (L-Val-L-Ile), cyclo (D-Val-L-Leu) and cyclo (L-Val-L-Leu). These six cyclic dipeptides increased the growth of *Bifidobacterium* species and had no effect on potentially harmful gut organisms. Moreover, supplementation with a mixture of these six cyclic dipeptides significantly increased the abundance of microorganisms related to the genus *Bifidobacterium* in a human colonic microbiota model culture system, although supplementation with a single type of dipeptide had no effect. These results show that cyclic dipeptides containing Val-Leu and Val-Ile produced by C-3102 could serve as bifidogenic growth factors in the gut microbial community.

## Introduction

In the past few decades, the human gut microbiota has been reported to play an important role in the promotion of health, and several illnesses are known to be influenced by imbalances of gut microbiota^1^. Therefore, manipulation of the gut microbiota is a clinical target for the treatment of gut microbiota-related diseases^2^. Bifidobacterial populations belonging to the phylum Actinobacteria are the most dominant microbial group present in the healthy infant gut^3^. The population of *Bifidobacterium* in the human gut decreases gradually from infancy to childhood, then remains relatively stable during an adult’s life, and ultimately decreases in old age^4^. Numerous health-promoting effects have been ascribed to certain strains of the genus *Bifidobacterium*, which are widely used as probiotics^5^. For instance, one Japanese study reported that allergic children had less bifidobacteria compared to non-allergic children at an early stage, 4 months of age, and prenatal supplementation of bifidobacteria to mothers and postnatal supplementation to infants reduced the risk of allergies^6^. It has also been reported that ingestion of *Bifidobacterium breve* has an anti-obesity effect in mice fed a high-fat diet^7^, and ingestion of *Bifidobacterium infantis* was effective in relieving symptom of irritable bowel syndrome in women^8,9^. These findings suggest an important role for bifiodobacteria in regulating intestinal homeostasis.

Some *Bacillus subtilis* strains are safe, survive passage through the human gastrointestinal tract, and do not induce any undesirable physiological effects in human subjects^10,11^. One reported probiotic effect of *B. subtilis* was associated with inhibiting the growth of pathogenic bacteria such as *Salmonella enterica* to improve the growth of beneficial bacteria such as lactobacilli^12^. In our previous study, *B. subtilis* C-3102 (hereinafter, referred to as C-3102) was tested using the TNO Gastro-Intestinal Model, simulating human conditions in the lumen of the gastrointestinal tract^13^. Administration of C-3102 increased the relative abundance of the genus *Bifidobacterium* and decreased that of the genus *Clostridium* in the large intestine model, after C-3102 spores germinated when passed through the stomach and the small intestine model. In addition, ingestion of C-3102 at 8.0 × 10^8^ colony forming units (cfu)/day for 8 days in human subjects increased the relative abundance of the genus *Bifidobacterium* and decreased the number of *Enterobacteriaceae*^14^. Recently, it has been reported that ingestion of C-3102 at 2.2 × 10^9^ cfu/day for 8 weeks improved loose stool symptoms in adults^15^, and ingesting 3.4 × 10^9^ cfu/day for 24 weeks increased the abundance of *Bifidobacterium* in postmenopausal women^16^. Therefore, C-3102 has the potential to modulate the gut microbiota to improve fecal properties in humans.

C-3102 is known to modulate gut microbiota, in particular, the genus *Bifidobacterium*; however, its mechanism is still unclear. It has been reported that many compounds, such as oligosaccharides, can serve as bifidogenic growth factors^17^. Since the production of bifidogenic growth factors by microorganisms has been reported^18^, C-3102 may also produce bifidogenic growth factors. Therefore, in this study we examined whether bifidogenic growth factors were produced by C-3102 as liquid soluble components.

## Results

### The identification of bifidogenic growth factors in culture supernatants of C-3102

**Figure 1:**
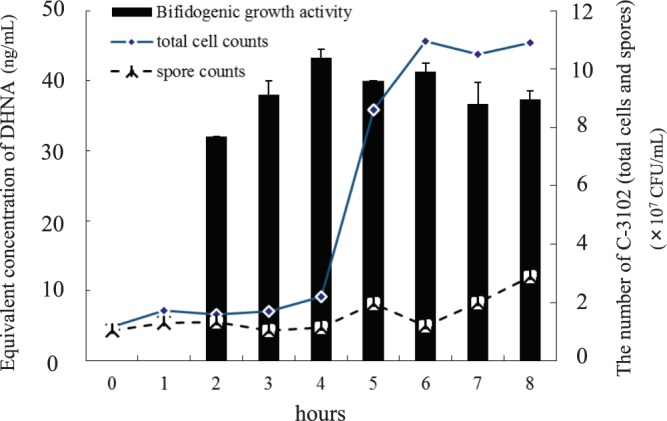
Total cell counts (diamonds) and spore counts (squares) of C-3102. C-3102 spores were inoculated in DSM improved medium. Bifidogenic growth activity (indicated by black bars) is reported as equivalent concentrations of DHNA, because high correlation (*r*^2^ = 0.993) was obtained between bifidogenic growth activity and the applied amount of DHNA from *Propionibacterium freudenreichii* culture^18^.

First, bifidogenic growth activity was investigated in cell-free culture supernatants of C-3102. C-3102 spores were inoculated in TPY medium^19^ and cultured with shaking at 37 **°**C. The culture supernatant was sampled every hour, and the total number of bacteria (germinated cells and spores) and the bifidogenic growth activity were measured. Soon after inoculation, C-3102 germinated and at 1 h of cultivation, the total cell counts were higher than the spore counts. Bifidogenic growth activity was seen from 2 hours after the germination of C-3102 spores, and showed a maximum value in 4–6 hours (Fig. 1). After that, the bifidogenic growth activity was maintained even if the culture time was prolonged. In this study, isolation and purification of bifidogenic growth factors were attempted using the culture supernatant at 6 hours, because total cell numbers saturated and relatively high bifidogenic activity was observed.

**Figure 2:**
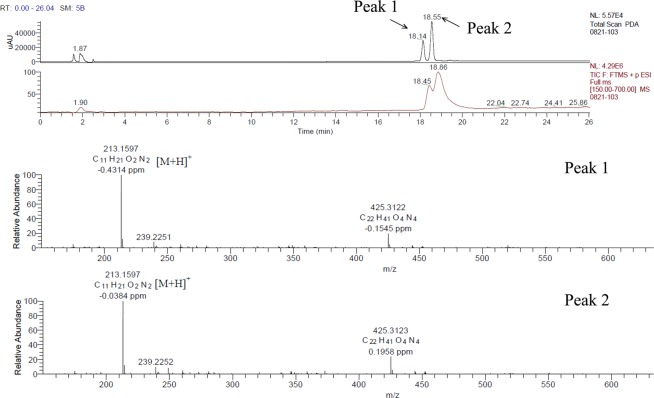
Analysis of culture samples from C-3102 by LC-MS/MS with Photodiode Array (PDA) and Total Ion Current (TIC). Upper graph: LC chromatograms. Two peaks detected at the retention times of 18.14 and 18.55 minutes were labeled peak 1 and peak 2, respectively. Lower graph: Mass spectra of peak 1 and peak 2. The molecular weight of the detected peak 1and peak 2 compounds was estimated to be 213.16.

**Figure 3:**
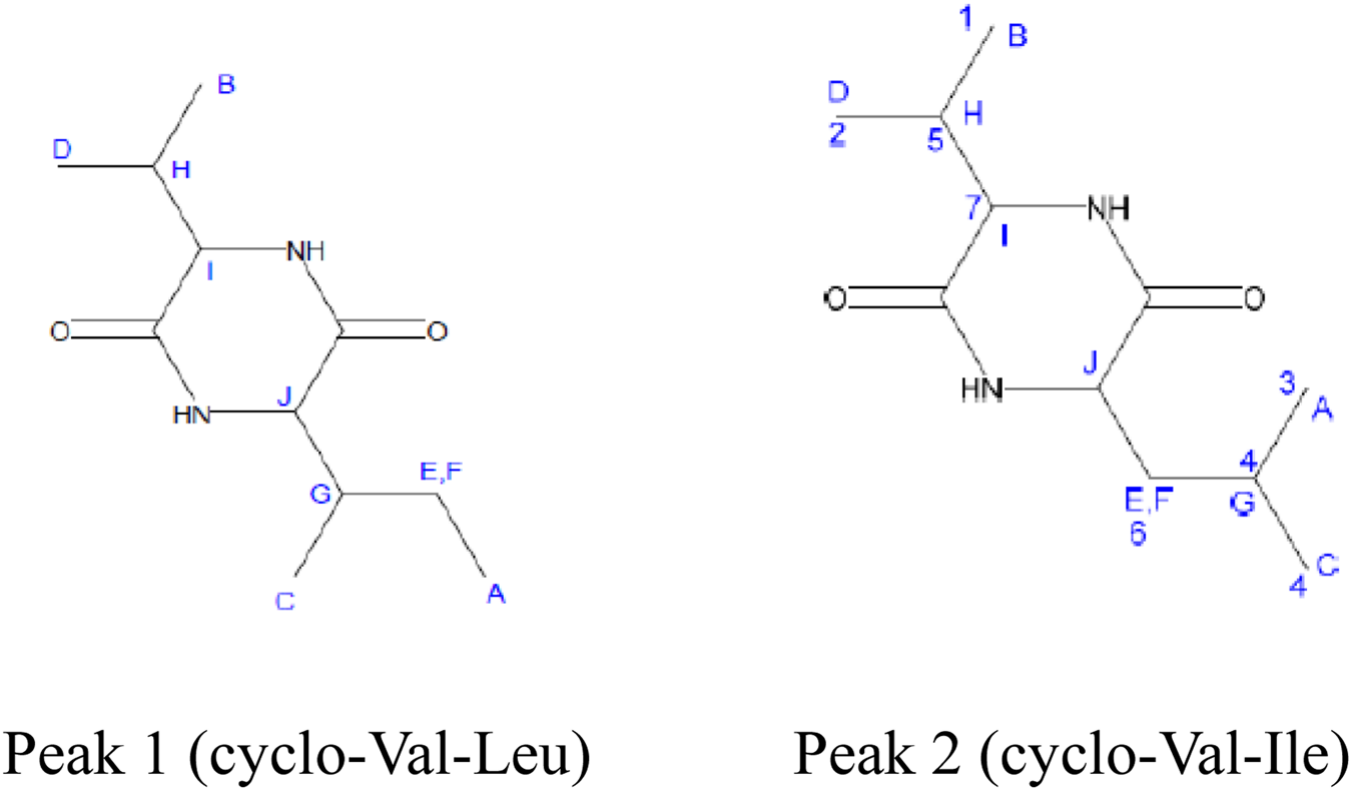
The structure of active compounds as analyzed by LC-MS/MS and LC-NMR. The numbers and letters labeling the compounds are based on the NMR results (Supplementary Tables 2 and 3).

A butanol extract of the C-3102 culture supernatant was prepared. The extract was further fractionated by high-performance liquid chromatography (HPLC), and a specific fraction having bifidogenic growth activity was obtained (Supplementary Fig. 1). In order to avoid the influence of amino acids and glucose, later eluting fractions having bifidogenic growth activity (Fractions 25, 26) were collected and subjected to liquid chromatography-tandem mass spectrometry (LC-MS/MS) (Fig. 2) and LC-nuclear magnetic resonance (NMR) to obtain information about the chemical structures of the components (Supplementary Tables 1–3). Two peaks were confirmed, which were predicted to be cyclo (Val-Leu) and cyclo (Val-Ile), respectively (Fig. 3).

### Chiral analysis of bifidogenic growth factors

The HPLC fraction of the cell-free culture supernatant of C-3102 with bifidogenic growth activity was hydrolyzed to obtain the amino acids present, and then the amino acids were analyzed by HPLC using a chiral column. By comparing the elution time between amino acids in the active fraction and standards, it was revealed that l-Val, d-Val, l-Ile, d-Ile and l-Leu were present, whereas d-Leu was not. Isoleucine has allo-isomers; however, chemically synthesized cyclic dipeptides containing allo-DL-Ile did not show bifidogenic growth activity on *Bifidobacterium adolescentis* (Supplementary Fig. 2). The results indicate that the bifidogenic growth factors present in the culture supernatant of C-3102 were cyclo (l-Val-l-Leu), cyclo (l-Val-l-Ile), cyclo (d-Val-d-Ile), cyclo (l-Val-d-Ile), cyclo (l-Val-d-Leu) and cyclo (d-Val-l-Ile).

### Effects of identified bifidogenic growth factors on bifidobacterial strains and harmful bacteria

**Figure 4:**
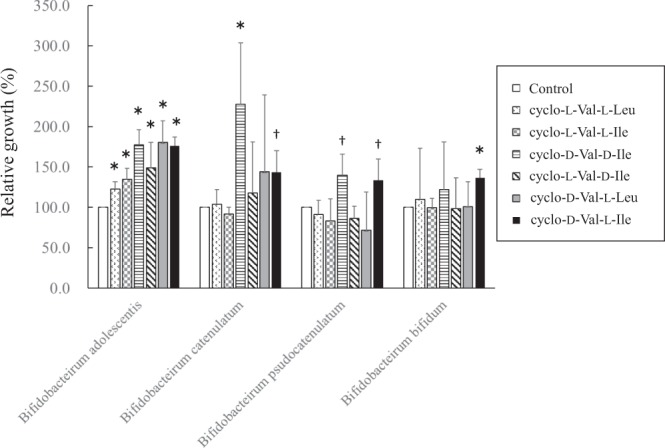
Growth activity of four *Bifidobacterium* species (*B. adolescentis*, *B. catenulatum*, *B. pseudocatenulatum* and *B. bifidum*). Each of the six bifidogenic growth factors was added at half the concentration in GAM broth and bacteria were cultivated in anaerobic conditions. Significant differences were determined by the Student t-test (*P < 0.05, ^†^*P* < 0.1). Error bars show the standard deviations of the mean.

In order to confirm that the chemically synthesized peptides, cyclo (l-Val-l-Leu), cyclo (l-Val-l-Ile), cyclo (d-Val-d-Ile), cyclo (l-Val-d-Ile), cyclo (l-Val-d-Leu) and cyclo (d-Val-l-Ile), stimulate only bifidobacterial growth and do not increase growth of harmful bacteria, the growth of representative bifidobacterial strains and harmful bacteria present in the human intestine was evaluated. Results showed that some of the cyclic dipeptides stimulated growth of bifidobacterial strains (*Bifidobacterium adolescentis*, *Bifidobacterium catenulatum*, *Bifidobacterium pseudocatenlatum*, and *Bifidobacterium bifidus*) in liquid culture (Fig. 4). Among the four *Bifidobacterium* species used in the experiment, *Bifidobacterium adolescentis* was particularly sensitive to the six growth factors. Regarding Bifidobacteria, the same growth activity was confirmed in solid culture as in liquid culture; however, the growth of harmful bacteria (*Clostridium perfringens*, *Escherichia coli*, *Bacteroides fragilis*, *Enterobacter aerogenes*, *Staphylococcus aureus*, *Enterococcus faecium*) was not stimulated (Supplementary Fig. 3 and Fig. 4). In particular, cyclo (D-Val-L-Ile) significantly stimulated the growth of three out of four *Bifidobacterium* species (*P* < 0.05, Dunnett test).

### ***Bifidobacterium*growth in the presence of fecal bacteria using an*in vitro*batch fermentation system**

**Figure 5:**
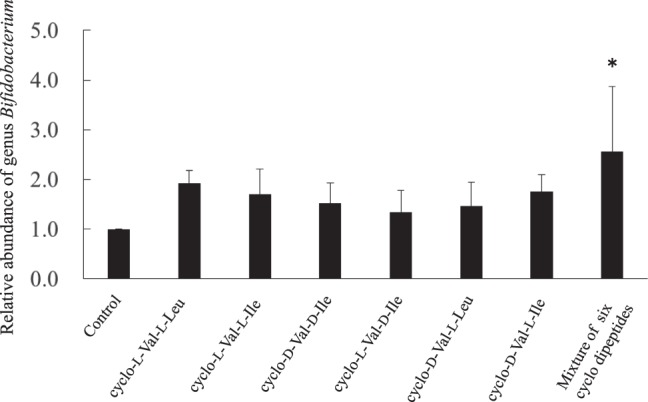
The relative abundance of *Bifidobacterium* in the *in vitro* human colonic microbiota model with 0.1% bifidogenic growth compounds. Fermentation was initiated by inoculating each of three human fecal samples, and bifidogenic growth compounds were added after 6 hours of incubation. After collecting the culture medium after 30 hours of incubation, DNA was extracted and quantitated using *Bifidobacterium* specific primers. To calculate the relative abundances, the 16S rRNA gene copy number of *Bifidobacterium* with each bifidogenic growth compound or the mixture was compared with that of the control without bifidogenic growth compound. Significant differences were determined by Dunnett test (*P < 0.05). Error bars show the standard deviation of the mean.

To confirm whether the six bifidogenic growth factors can increase *Bifidobacterium* numbers in the human colon where many microorganisms coexist, the proliferation effect was evaluated using a model culture system for the *in vitro* human colonic microbiota^20^. This human colonic microbiota model maintained the number and richness of bacterial species in human feces, and thus is effective for investigating the effect of dosage compounds on human colonic microbiota^20^. A model culture system was operated by adding one or a mixture of cyclo (L-Val-L-Leu), cyclo (L-Val-L-Ile), cyclo (D-Val-D-Ile), cyclo (L-Val-D-Ile), cyclo (L-Val-D-Leu), and/or cyclo (D-Val-L-Ile) at 0.1% (1000 ppm). The amounts of the cyclic peptides were set to 1000 ppm, because the GAM medium for the model culture system contained approximately 200 ppm of these cyclic peptides. A model culture system that included none of the above compounds was also prepared as a control. Each of three human fecal samples was used as the inoculum. Addition of each of the six cyclic dipeptides or the mixture suggested that *Bifidobacterium* numbers increased compared with the control group (Fig. 5). In particular, the addition of a mixture of these six compounds significantly increased *Bifidobacterium* numbers in the human colonic microbiota model. In response to the addition of the mixture, the relative abundances of bacteria related to the genus *Bifidobacterium* increased in two microbiota models out of three (2.98%→7.73%, 8.56%→7.67% and 0.06%→0.24%, for F45, M41 and M38, respectively), when the relative abundances of the genus *Bifidobacterium* were relatively low (Supplementary Fig. 5). However, using next generation sequencing analysis, no significant differences were found in the relative ratios of all the bacterial species in response to the addition of the mixture (Supplementary Table 4). The consumption of bifidogenic growth factors was calculated by comparing the initial and final concentrations in the culture medium. When one kind of bifidogenic growth factor was added into the human colonic microbiota model, it was not consumed (Table 1). However, when the mixture of six factors was added, a portion of the bifidogenic growth factors tended to be consumed (*P* = 0.07, paired t-test). These results indicate that the mixture of bifidogenic growth factors could stimulate the growth of *Bifidobacterium* spp. in the human colonic microbiota model.

**Table 1:** Consumption of bifidogenic growth compounds in the *in vitro* culture system of the human colonic microbiota model.

	Relative ratios of bifidogenic growth factors	p-value
Control	1.15 ± 0.20	0.30
l-Val-L-Leu	1.05 ± 0.17	0.66
l-Val- L-Ile	1.06 ± 0.26	0.72
D-Val- D-Ile	0.98 ± 0.07	0.67
L-Val- D-Ile	1.14 ± 0.20	0.28
D-Val- L-Leu	1.20 ± 0.19	0.15
D-Val- L-Ile	1.01 ± 0.07	0.88
Mixture of six cyclic dipeptides	0.91 ± 0.04	0.07

Changes are presented as the ratios of the final concentrations in the model culture system relative to those at the initial time of bifidogenic growth factor addition. Standard deviations of the mean values are shown, and the p values were determined by the paired t-test (vs initial concentration, n = 3).

## Discussion

Bifidogenic growth factors can function as prebiotics to modulate the composition of human colonic microbiota. We found a mixture of six cyclic dipeptides [cyclo (L-Val-L-Leu), cyclo (L-Val-L-Ile), cyclo (D-Val-D-Ile), cyclo (L-Val-D-Ile), cyclo (L-Val-D-Leu) and cyclo (D-Val-L-Ile)] that were produced by C-3102 and functioned as bifidogenic growth factors. Some of the cyclic dipeptides had the ability to selectively stimulate the growth of *Bifidobacterium* strains and showed no effect on harmful bacteria. Moreover, the mixture of these six dipeptides had the potential to stimulate the growth of microorganisms related to genus *Bifidobacterium* even in the human colon, which is expected to harbor more than 1000 microbial species^21^. In previous studies, an intermediary metabolite of menaquinone biosynthesis, 1,4-dihydroxy-2-naphthoic acid (DHNA) produced by *Propionibacterium freudenreichii*, was shown to function as a bifidogenic growth factor^18^ and an inhibitor of the harmful bacterium *Helicobacter pylori*^22^. Here, the mixture of six cyclic dipeptides was identified as new bifidogenic growth factor. The mixture of six cyclic dipeptides was more effective to stimulate *Bifidobacterium* spp. in the human colon than single cyclic dipeptides, probably because the human-resident bifidobacterial community contains multiple species^23^ and each cyclic dipeptide may have different effects on each *Bifidobacterium* sp.

*B. subtilis* strains are widely distributed in soil where they compete with pathogens and stimulate plant growth^24^. They are known to produce a range of secondary metabolites, including polyketides and peptides^24^. For example, *Bacillus* spp. secrete a dipeptide known as bacilysin, synthesized from L-alanine and L-anticapsin^25^. Peptide formation in microorganisms is generally carried out by the ribosomal system, but other enzymatic machineries, such as non-ribosomal peptide synthetases, have been found in members of the *B. subtilis* group^26^. We showed the production of cyclic dipeptides containing Val-Leu and/or Val-Ile by C-3102. It was found that D-isomers were present in the culture supernatant of C-3102, although D-form amino acids were not included in the medium before cultivation. Since nine racemases are encoded in the genome of strain C-3102, there is a possibility that D-form amino acids were produced by the action of these enzymes.

To date, there are no other reports that cyclic dipeptides containing Val-Leu and/or Val-Ile stimulate the growth of *Bifidobacterium* spp. and it is not clear at this time how these cyclic dipeptides affect *Bifidobacterium* spp. It is possible that cyclic dipeptides might be metabolized by *Bifidobacterium* spp. to increase growth. A dipeptidase enzyme was purified from *Bifidobacterium longum* BORI^27^, so at least this strain is known to have an enzyme necessary for cleavage of dipeptides to the component amino acids. In addition, bifidobacteria have a high demand for essential growth factors, such as various amino acids, and supplementation with branched-chain amino acids (such as Val, Leu, and Ile) increased *Bifidobacterium* spp. in the gut microbiota of mice^28^. However, careful interpretation is necessary, because although a mixture of cyclic dipeptides increased the abundance of *Bifidobacterium* spp., most of the compounds were not consumed.

Reportedly, the protein lactoferrin present in the milk of most mammals has various biological functions such as antimicrobial activity and bifidogenic activity and may stimulate the incorporation of nutrients from the environment^29^. Bifidobacteria have a lactoferrin-binding protein on the cell surface^29^. Thus, there is a possibility that a binding protein that recognizes the specific peptide sequence of these six cyclic dipeptides may be present on the surface of *Bifidobacterium* species. It is also known that the diketopiperazine structure contained in the cyclic dipeptide is a quorum sensing signal molecule^30,31^. Degrassi G. *et al*. reported that the diketopiperazine L-Val-L-Leu produced by *Pseudomonas putida* WCS358 was detected by LuxR and may be capable of cross talk with the LuxR-LuxI quorum sensing system^31^. In addition, *Bifidobacterium longum* NCC2705 was reported to have LuxS homologs that function in the production of autoinducer-2 (AI-2), and biofilm formation by *B. longum* NCC2705 was enhanced by supplementation of with AI-2^32^. Since an increase in bacterial cell population density is concomitant with an increase in the concentration of signal molecules in the extracellular environment^33^, the bifidogenic growth factors identified in this study may play a role in quorum sensing to increase the proliferation of Gram-positive *Bifidobacterium* spp.

## Materials and Methods

### **Culture conditions, extract preparation and growth measurement of*B. subtilis*C-3102**

C-3102 was inoculated in Difco sporulation medium (DSM) improved medium (1% Glucose, 0.5% Casamino acids, 0.01% KCl, 0.00122% MgSO_4_, pH 7.6), and cultured for 6 h at 37°C. A portion of the C-3102 culture solution was collected and subjected to centrifugation at 10,000 × g for 15 min to remove C-3102 cells. An equal volume of butanol was added to the supernatant. The butanol extract was collected after shaking for 5 min at room temperature and evaporated to dryness. The residue was dissolved in ethanol and used as the extract sample.

To monitor growth of C-3102, ten-fold dilutions of the culture were made using saline solution. The diluted samples were directly inoculated onto Trypticase Soy Broth (BBL-211768; BD, Franklin Lakes, NJ, USA) medium solidified with 2% agar. After aerobic incubation at 37 °C for 24 h, colonies were counted to determine the total cell counts. To measure spore counts only, the diluted solutions were heated at 65 °C for 35 min and then cooled on ice for 10 min. After inoculation onto the above agar plate, heated samples were incubated aerobically to allow formation of colonies, as described above.

### Assay of bifidogenic growth activity

*Bifidobacterium adolescentis*^T^ [Japan Collection of Microorganisms (JCM) 1275] was precultured in Gifu Anaerobic Medium (GAM) broth (Nissui Pharmaceutical, 05422). TPY agar (3% glucose, 0.4% Trypticase peptone, 0.15% Proteose peptone No. 3, 0.25% Yeast extract, 0.2% K_2_HPO_4_, 0.3% KH_2_PO_4_, 0.025% MgCl_2_·6H_2_O, 0.025% L-cysteine, 0.0005% FeSO_4_·7H_2_O, 1.5% agar) was prepared by autoclaving at 121 °C for 15 min. Then, the TPY agar was cooled at 50 °C, poured into petri dishes (diameter: 10 cm), and dried until no water drops were visible.

C-3102 cultures were subjected to centrifugation at 20,000 × g for 5 min to remove cells and collect the supernatant. These supernatants were filtered through a 0.20 μm mixed cellulose ester membrane (ADVANTEC Co., Ltd., Tokyo, Japan), and 60 μL of filtrate was spotted onto an 8 mm sterile paper disc. The paper disc was placed on the TPY agar plate inoculated with 0.1–0.2% (v/v) *B. adolescentis* and the plates were then incubated anaerobically at 37 °C for 18 h using a GasPak anaerobic system (Becton, Dickinson and Company, Franklin Lakes, NJ, USA). The growth diameter of *B. adolescentis* was measured directly after incubation, and the average size was calculated from three replicates. The growth diameter obtained using 1,4-dihydroxy-2-naphthoic acid (DHNA) dissolved in ethanol (1, 10 and 100 ng/mL) was used as a standard^18^. Thus, bifidogenic growth activity was shown as the corresponding amount of DHNA to obtain the same growth diameter.

At the same time, for each C-3102 culture solution collected, the OD_210_ was measured as an index of the amino acid concentration. The glucose concentration was measured with the LabAssay^TM^ Glucose (FUJIFILM Wako Pure Chemical Corporation, Osaka, Japan).

### Sample fractionation using HPLC for identification of bifidogenic growth factors

For fractionation of butanol extracts, samples were loaded onto an Atlantic T3 column (150 × 4.6 mm i.d., 5 μm particle size) (Waters Corporation, Milford, MA, USA) on a Prominence HPLC unit (Shimadzu Cooperation, Kyoto, Japan). The mobile phase employed a MilliQ water (as buffer A) and acetonitrile (as buffer B). A column temperature of 40 °C was used. The elution was carried out using a gradient of 10% buffer B for 0.5 min and 10–50% buffer B for 40 min, at a flow rate of 1.0 mL/min. The eluted fractions were monitored at a wavelength of 210 nm using a UV detector. Fractions were collected every minute.

### LC-MS/MS

The LC-MS/MS analysis was carried out using a LTQ-Orbitrap Discovery mass spectrometry system (Thermo Fisher Scientific Inc., Waltham, MA, USA). Chromatographic separation was performed using a common ODS column (150 × 46 mm i.d., 5 μm particle size, Sumika Chemical Analysis Service, Ltd., Tokyo, Japan). The mobile phases employed MilliQ water (as buffer A) and acetonitrile (as buffer B). A column temperature of 40 °C was used. The elution was carried out using a gradient of 10% buffer B for 0.5 min and 10–50% buffer B for 40 min, at a flow rate of 1.0 mL/min. A PDA detector was connected in front of the mass detector. An electrospray ionization (ESI) source was operated for MS measurement, and the mass range was set to optically pass ions from *m/z* 150–700.

### LC-NMR

The LC-NMR spectra were acquired on an AVANCE II 800US2 (Bruker BioSpin K.K., Kanagawa, Japan) coupled to an Agilent 1200 (Agilent Technologies, Inc., Santa Clara, CA, USA). Chromatographic separation was performed on a common ODS column (150 × 46 mm i.d., 5 μm particle size, Sumika Chemical Analysis Service, Ltd., Tokyo, Japan) using a two solvent gradient system (A: Deuterated water; B: acetonitrile). The elution was carried out using a gradient of 10% buffer B for 0.5 min and 10–50% buffer B for 40 min, at a flow rate of 1.0 mL/min. The resonance frequency was 800 MHz, and measurement modes were ^1^H NMR, ^13^C NMR, ^13^C-^1^H COSY and HMBC. The complete assignments of the ^1^H and ^13^C NMR chemical shifts were obtained by means of 2D NMR techniques, including ^13^C-^1^H COSY and HMBC spectra.

### Chiral HPLC analysis

The HPLC system consisted of a Prominence unit (Shimadzu Cooperation, Kyoto, Japan), a SUMICHIRAL OA-5000 column (150 × 4.6 mm i.d., 5 μm particle size) (Sumika Chemical Analysis Service, Ltd., Tokyo, Japan), and UV detector (Shimadzu Cooperation, Kyoto, Japan). The mobile phase was a mixture of 0.2 mM copper sulfate and acetonitrile.

### **Growth activity of*Bifidobacterium*species by liquid culture**

*Bifidobacterium adolescentis* ^T^ (JCM1275), *Bifidobacterium catenulatum* (JCM1194), *Bifidobacterium pseudocatenulatum* (JCM1200), and *Bifidobacterium bifidum* (JCM1255) were used as representative *Bifidobacterium* species.

Each of the six bifidogenic growth compounds [cyclo (L-Val-L-Leu), cyclo (L-Val-L-Ile), cyclo (D-Val-D-Ile), cyclo (L-Val-D-Ile), cyclo (L-Val-D-Leu), cyclo (D-Val-L-Ile), obtained from PEPTIDE INSTITUTE, INC.] was dissolved in ethanol and added to a final concentration half that in GAM broth (100 ppm final concentration). Ethanol was added as a negative control. The broth was inoculated with 1.0% (v/v) precultured bacteria and incubated anaerobically at 37 °C for 14–16 h. After incubation, the OD_600_ of each suspension was determined.

### Growth activity of gut bacteria by solid culture

*Bifidobacterium adolescentis* ^T^ (JCM1275), *Bifidobacterium catenulatum* (JCM1194), *Bifidobacterium pseudocatenulatum* (JCM1200), and *Bifidobacterium bifidum* (JCM1255) were used as representative *Bifidobacterium* species. *Clostridium perfringens* ^T^ (JCM1290), *Escherichia coli* ^T^ (JCM1649), *Bacteroides fragilis* ^T^ (JCM11019), *Enterobacter aerogenes* ^T^ (JCM5804), *Staphylococcus aureus* ^T^ (JCM1235), and *Enterococcus faecium* ^T^ (JCM20624) were selected as harmful bacteria. These bacteria were precultured in TPY medium anaerobically at 37 °C for 18 h.

Each of the six bifidogenic growth compounds [cyclo (L-Val-L-Leu), cyclo (L-Val-L-Ile), cyclo (D-Val-D-Ile), cyclo (L-Val-D-Ile), cyclo (L-Val-D-Leu), cyclo (D-Val-L-Ile), obtained from PEPTIDE INSTITUTE, INC.] was dissolved in ethanol and added to TPY agar poured in 10 cm dishes (0.1 ppm final concentration). Ethanol was added as negative control. The agar plates were inoculated with 1.0% (v/v) precultured bacteria, and incubated anaerobically at 37 °C for 48 h. After incubation, the colonies of the evaluated bacterial species were collected and suspended in 5 mL of distilled water. The OD_600_ of each suspension was determined.

### Fecal sample collection from human volunteers

Fecal samples were obtained from three healthy Japanese human volunteers (F45, M41, and M38). The experimental contents and methods were explained to all subjects and written informed consent was obtained. Fecal samples were immediately collected using an anaerobic culture swab (212550 BD BBL Culture Swab; Becton, Dickinson and Company, Franklin Lakes, NJ, USA) and used within 24 h. The study was performed in accordance with the principles of the Declaration of Helsinki and the guidelines of our institution and approved by the institutional ethics review board at Kobe University (research code; 1902, approval date, May 10, 2016). The methods used to collect fecal samples and operate the model culture system were performed in accordance with the approved guidelines of the Medical Ethics Committee at Kobe University.

### Operation of the model culture system with and without bifidogenic growth factors

The model culture system was operated using a multi-channel fermenter (Bio Jr.8; ABLE, Tokyo, Japan), which consists of eight parallel and independent vessels (working volume, 100 mL), as described previously^20^. In short, each vessel contained autoclaved Gifu Anaerobic Medium (GAM; Nissui Pharmaceutical Co., Ltd., Tokyo, Japan). Anaerobic condition in the vessel was achieved by purging with a filter-sterilized mixture of N_2_ and CO_2_ (80:20; 15 mL/min). To prepare the inoculum, the fecal sample in the swab was suspended in 2.0 mL of 0.1 M phosphate buffer (pH 6.5, consisting of 0.2 M NaH_2_PO_4_ and 0.1 M Na_2_HPO_4_) supplemented with 1.0% L-ascorbic acid (Wako Pure Chemical Industries, Osaka, Japan).

Cultivations were initiated by inoculating one fecal suspension (100 µL) into each vessel. During fermentation at 37 °C, the culture broth was stirred at 300 rpm with a magnetic stirrer and continuously purged with a filter-sterilized mixture of gas to maintain anaerobic conditions. Aliquots (1 mL) of culture broth were collected. Feces and culture broth samples were stored at ‒20 °C until use.

To evaluate the effect of bifidogenic growth factors, each type of bifidogenic growth factor or a mixture of factors was added into one of the vessels at a final concentration of 0.1% at 6 hours after the initiation of fermentation. The following bifidogenic growth factors were used: cyclo (L-Val-L-Leu), cyclo (L-Val-L-Ile), cyclo (D-Val-D-Ile), cyclo (L-Val-D-Ile), cyclo (L-Val-D-Leu), cyclo (D-Val-L-Ile) and the mixture of these six bifidogenic growth factors. A control vessel without bifidogenic growth factors was prepared.

### Extraction of microbial genomic DNA

Microbial genomic DNA was extracted from suspended feces and culture broth from the model culture system after 30 h of fermentation, as described previously^34^. Purified DNA was eluted into TE buffer (10 mM Tris HCl, 1.0 mM EDTA) and stored at −20 °C until use.

### Real-time PCR

The number of *Bifidobacterium* was estimated using 16S rRNA gene-targeted primers Bif-F (5′-TCGCGTCYGGTGTGAAAG-3′) and Bif-R (5′-CCACATCCAGCRTCCAC-3′)^35^. Real-time PCR was performed in triplicate using a LightCycler 480 System II real-time PCR system with LightCycler 480 software Service Pack 4 (Roche Diagnostics, Tokyo, Japan). Amplification and detection were carried out in 96-well plates with SYBR Green I. The amplification program used is as follows: 94 °C for 30 s, 56 °C for 20 s, 72 °C for 30 s; 35 cycles. The cycle threshold of each sample was compared to standard curves made by diluting genomic DNA (10-fold serial dilution) from cultures of *Bifidobacterium adolescentis* JCM1275. Melting-point-determination analysis allowed confirmation of the specificity of the amplification products. PCR efficiencies were higher than 85%.

### **Analysis of the composition of the*in vitro*human colonic microbiota model by next generation sequencing**

Microbiota composition was determined with a MiSeq V2 kit (Illumina, San Diego, CA, USA), as described previously^15^.

### Measurement of bifidogenic growth factors in the model culture system

For the LC-MS/MS procedure, we employed a Nexera HPLC system (Shimazu, Kyoto, Japan) and an API6500^TM^ triple quadrupole mass spectrometer (Sciev, Framingham, MA, USA). Analyst software was used to control the instruments and to process all recorded data. Chromatographic separation was performed using a CHIRAL PAK AS-3R (150 mm × 4.6 mm i.d., 5 μm particle size) (Daicel Cooperation, Osaka, Japan), and the mobile phase for separation was 99% acetonitrile. A column temperature of 25 °C was used, along with a sample injection volume of 5 μL. An electrospray ionization (APCI) source was operated for MS measurements, and the measurement range was *m/z* 200–220. Sample preparation was performed with reference to the method described in Kakitani *et al*.^36^; briefly, a 1 mL sample was placed in a 15 mL polypropylene centrifuge tube, and 2 mL of acetonitrile was added. The contents of the Supel QuE non-buffered tube were then added to the sample solution, and the mixture was shaken for 5 min prior to centrifugation for 3 min at 350 rpm to obtain the extract. A sample of the extract (i.e., the upper acetonitrile layer, 1 mL) was then transferred to the Supel QuE PSA SPE clean-up tube. The tube was then vortexed for 30 sec and centrifuged for 1 min at 3500 rpm. The resulting supernatant was passed through a PTEE filter and analyzed by LC-MS/MS.

### Statistical analyses

Statistical test was performed using SPSS ver. 25.0 software (IBM Japan, Ltd., Tokyo, Japan). *P* < 0.05 was considered statistically significant.

## Supplementary information

Supplementary information.

Dataset 1.

## References

[1] Marchesi JR (2016). The gut microbiota and host health: a new clinical frontier. Gut.

[2] Cammarota G, Ianiro G, Bibbò S, Gasbarrini A (2014). Gut microbiota modulation: probiotics, antibiotics or fecal microbiota transplantation?. Intern. Emerg. Med..

[3] Arboleya S, Watkins C, Stanton C, Ross RP (2016). Gut bifidobacteria populations in human health and aging. Front. Microbiol..

[4] Odamaki T (2016). Age-related changes in gut microbiota composition from newborn to centenarian: a cross-sectional study. BMC Microbiol..

[5] Sarkar A, Mandal S (2016). Bifidobacteria-Insight into clinical outcomes and mechanisms of its probiotic action. Microbiol. Res..

[6] Enomoto T (2014). Effects of bifidobacterial supplementation to pregnant women and infants in the prevention of allergy development in infants and on fecal microbiota. Allergol. Int..

[7] Kondo S (2010). Antiobesity effects of *Bifidobacterium breve* strain B-3 supplementation in a mouse model with high-fat diet-induced obesity. Biosci. Biotechnol. Biochem..

[8] Whorwell PJ (2006). Efficacy of an encapsulated probiotic Bifidobacterium infantis 35624 in women with irritable bowel syndrome. Am. J. Gastroenterol..

[9] Tojo R (2014). Intestinal microbiota in health and disease: role of bifidobacteria in gut homeostasis. World J. Gastroenterol..

[10] Lefevre M (2017). Safety assessment of Bacillus subtilis CU1 for use as a probiotic in humans. Regul. Toxicol. Pharmacol..

[11] Hanifi A (2015). Evaluation of *Bacillus subtilis* R0179 on gastrointestinal viability and general wellness: a randomized, double-blind, placebo-controlled trial in healthy adults. Benef. Microbes..

[12] Horie M, Koike T, Sugino S, Umeno A, Yoshida Y (2018). Evaluation of probiotic and prebiotic-like effect of *Bacillus subtilis* BN on growth of lactobacilli. J. Gen. Appl. Microbiol..

[13] Hatanaka M (2012). Influence of *Bacillus subtilis* C-3102 on microbiota in a dynamic *in vitro* model of the gastrointestinal tract simulating human condition. Benef. Microbes.

[14] Suzuki, H. *et al*. Effect of *Bacillus subtilis* C-3102 intakes on the composition and metabolic activity of fecal microflora of humans. *Chounai Saikingaku Zasshi* **18**, 93–99 (2004).

[15] Hatanaka M (2018). Effect of *Bacillus subtilis* C-3102 on loose stools in healthy volunteers. Benef. Microbes.

[16] Takimoto T (2018). Effect of *Bacillus subtilis* C-3102 on bone mineral density in healthy postmenopausal Japanese women: a randomized, placebo-controlled, double-blind clinical trial. Biosci Microbiota Food Health..

[17] Al-Sheraji SH (2013). Prebiotics as functional foods: a review. J. Funct. Foods..

[18] Isawa K (2002). Isolation and identification of a new bifidogenic growth stimulator produced by *Propionibacterium freudenreichii* ET-3. Biosci. Biotechnol. Biochem..

[19] Yang X (2005). Feruloyl oligosaccharides stimulate the growth of *Bifidobacterium bifidum*. Anaerobe..

[20] Sasaki D (2018). Low amount of dietary fibre increase *in vitro* production of short-chain fatty acids without changing human colonic microbiota structure. Sci. Rep..

[21] Sasaki K (2019). Construction of a model culture system of human colonic microbiota to detect decreased *Lachnospiraceae* abundance and butyrogenesis in the feces of ulcerative colitis patients. Biotechnol. J..

[22] Nagata K (2010). The bifidogenic growth simulator inhibits the growth and respiration of *Helicobacter pylori*. Helicobacter..

[23] Wong CB, Sugahara H, Odamaki T, Xiao JZ (2018). Different physiological properties of human-residential and non-human-residential bifidobacteria in human health. Benef. Microbes..

[24] Harwood CR, Mouillon JM, Pohl S, Arnau J (2018). Secondary metabolite production and the safety of industrially important members of the *Bacillus subtilis* group. FEMS Microbiol. Rev..

[25] Shomura Y (2012). Structural and enzymatic characterization of BacD, an L-amino acid dipeptide ligase from *Bacillus subtilis*. Protein Sci..

[26] Yagasaki M, Hashimoto S (2008). Synthesis and application of dipeptide; current status and perspectives. Appl. Microbiol. Biotechnol..

[27] Seo JM, Ji GE, Cho SH, Park MS, Lee HJ (2007). Characterization of Bifidobacterium longum BORI dipeptidase belonging to the U34 family. Appl. Environ. Microbiol..

[28] Yang Z (2016). Metabolic shifts and structural changes in the gut microbiota upon branched-chain amino acid supplementation in middle-aged mice. Amino acids..

[29] Oda H (2013). Isolation of a bifidogenic peptide from the pepsin hydrolysate of bovine lactoferrin. Appl. Environ. Microbiol..

[30] Daniels R, Vanderleyden J, Michiels J (2004). Quorum sensing and swarming migration in bacteria. FEMS Microbiol. Rev.

[31] Degrassi G (2002). Plant growth-promoting Pseudomonas putida WCS3358 produces and secretes four cyclic dipeptides: Cross-talk with quorum sensing bacterial sensors. Curr. Microbiol..

[32] Sun Z (2014). Bifidobacteria exhibit LuxS-dependent autoinducer 2 activity and biofilm formation. PLoS One..

[33] Atkinson S, Williams P (2009). Quorum sensing and social networking in the microbial world. J. R. Soc. Interface..

[34] Takagi R (2016). A single-batch fermentation system to simulate human colonic microbiota for high-throughput evaluation of prebiotics. PLoS One.

[35] Rintilla T (2004). Development of an extensive set of 16S rDNA-targeted primers for quantification of pathogenic and indigenous bacteria in feacal samples by real-time PCR. J. Appl. Microbiol..

[36] Kakitani A (2017). A rapid and sensitive analysis of dithiocarbamate fungicides using modified QuEChERS method and liquid chromatography-tandem mass spectrometry. J. Pestic Sci..

