# Supplementary material for: Effective bifidogenic growth factors cyclo-Val-Leu and cyclo-Val-Ile produced by Bacillus subtilis C-3102 in the human colonic microbiota model
Source: Sci Rep. 2020 May 5;10:7591. doi: 10.1038/s41598-020-64374-w (PMC7200657; doi:10.1038/s41598-020-64374-w)
Supplement: Supplementary file 1 — Supplementary information. [file 41598_2020_64374_MOESM1_ESM.pdf]

Supplementary information

Title

Effective bifidogenic growth factors cyclo-Val-Leu and cyclo-Val-Ile produced by *Bacillus subtilis* C-3102 in the human colonic microbiota model

Misaki Hatanaka[1], Hiroto Morita[2], Yumi Aoyagi[1], Kengo Sasaki[3], Daisuke Sasaki[3], Akihiko Kondo[3] and Teppei Nakamura[2]

[1]Research & Development Dept, Asahi Calpis Wellness Co., Ltd., 4-1, 2-chome, Ebisu-Minami, Shibuya-ku, Tokyo, 150-0022, Japan.

[2]Department of Microbiological Flora Technology, Core Technology Laboratories, Asahi Quality and Innovations Co., Ltd. 5-11-10 Fuchinobe, Chuo-ku, Sagamihara-shi 252-0206, Japan.

[3]Graduate School of Science, Technology and Innovation, Kobe University, 1-1 Rokkodai-cho, Nada-ku, Kobe, Hyogo 657-8501, Japan.

Corresponding author*

Misaki Hatanaka

Research & Development Dept., Asahi calpis wellness Co., Ltd.

4-1, 2-Chome, Ebisu-Minami, Shibuya-ku, Tokyo, 150-0022, Japan

+81-3-6412-3256 (TEL), +81- 3-5721-4036 (FAX)

misaki.hatanaka@asahicalpis-w.co.jp

Supplementary Table 1.

Speculated composition of bifidogenic growth factors in the culture supernatant of C-3102. For peak 1 and peak 2, the molecular weight was estimated from the LC-MS results, and the structure was predicted from the ion fragments obtained from LC-MS / MS.

Peak	MS detection ion (m/z)	MS/MS detection ion (m/z)	Speculated composition
Peak 1	213.1597 $C_{11}H_{21}O_2N_2$ [M+H]+	185.1646	$C_{10}H_{21}ON_2$
		168.1381	$C_{10}H_{18}ON_2$
		86.0963	$C_5H_{12}N$
		72.0868	$C_4H_{10}N$
Peak 2	213.1597 $C_{11}H_{21}O_2N_2$ [M+H]+	185.1647	$C_{10}H_{21}ON_2$
		168.1381	$C_{10}H_{18}ON_2$
		86.0963	$C_5H_{12}N$
		72.0806	$C_4H_{10}N$

Supplementary Table 2.

The results of Peak 1 analyzed by NMR

^{1}H NMR			
Signal	Chemical shift values (ppm)	multiplicity	The number of proton
A	0.77	triplet	3
B	0.78	doublet	3
C	0.89	doublet	3
D	0.90	doublet	3
E	1.08	multiplet	1
F	1.31	multiplet	1
G	approximately 2	-	-
H	2.14	multiplet	1
I	3.80	double doublet	1
J	3.86	double doublet	1

Supplementary Table 3.

The results of Peak 2 analyzed by NMR.

^{13}C NMR		^{1}H NMR				
No	Chemical shift values (ppm)	No	Chemical shift values (ppm)	multiplicity	multiplicity	HMBC correlation
1	approximately 17	B	0.80	doublet	3	D
2	approximately 18	D	0.89	doublet	3	B
3	approximately 20	A	0.80	doublet	3	C
4	approximately 23	C<br>G	0.82<br>1.66	doublet<br>multiplet	3<br>1	A,C
5	approximately 32	H	2.08	multiplet	1	B,D
6	approximately 44	E<br>F	1.54<br>1.58	multiplet<br>multiplet	1<br>1	A,C
7	approximately 60	I	3.76	double doublet	1	B,D
-	-	J	3.92	double double doublet	1	-

Supplementary Table 4.

Composition of microbiota in the human colonic microbiota model

Fermentation was initiated by inoculating each of three human fecal samples. A mixture of six bifidogenic growth compounds was added after 6 hours of incubation and no addition was used as the control. After collecting the culture medium after 30 hours of incubation, the microbiota composition was determined by next generation sequencing. Standard deviations of the mean values are shown for each relative abundance of bacteria.

Phylum	Family	Genus	Relative abundance (%)					
			Control			Mixture of six cyclo peptides		
Actinobacteria	*Bifidobacteriaceae*	*Bifidobacterium*	3.87	±	4.32	5.22	±	4.31
Bacteroidetes	*Bacteroidaceae*	*Bacteroides*	25.23	±	16.59	21.74	±	13.29
Firmicutes	*Enterococcaceae*	*Enterococcus*	7.95	±	10.62	8.31	±	8.14
	Lachnospiraceae	*unkown*	5.09	±	2.52	6.69	±	3.93
		Blautia	3.28	±	2.31	1.84	±	1.04
		Coprococcus	1.13	±	0.57	2.41	±	2.68
		Dorea	1.13	±	1.34	0.97	±	0.79
		[Ruminococcus]	1.43	±	0.93	3.09	±	2.24
	Peptostreptococcaceae	*[Clostridium]*	3.06	±	2.78	0.00	±	0.00
	Ruminococcaceae	*Faecalibacterium*	2.04	±	1.91	1.56	±	1.45
		Oscillospira	0.68	±	0.97	1.24	±	2.03
	Veillonellaceae	*Dialister*	0.74	±	0.73	0.90	±	0.79
		Phascolarctobacterium	1.27	±	1.30	0.92	±	0.96
	[Mogibacteriaceae]	*Mogibacterium*	0.43	±	0.74	1.18	±	2.05
	Erysipelotrichaceae	*unknown*	0.76	±	1.30	0.36	±	0.62
		Clostridium	3.08	±	3.64	2.74	±	3.68
		[Eubacterium]	2.02	±	3.34	0.85	±	1.27
		Fusobacterium	4.77	±	8.26	4.90	±	8.49
Proteobacteria	*Alcaligenaceae*	*Sutterella*	0.51	±	0.57	0.42	±	0.45
	Enterobacteriacea	*Other*	8.30	±	12.92	9.46	±	14.58
		Escherichia	12.91	±	5.74	14.05	±	6.60

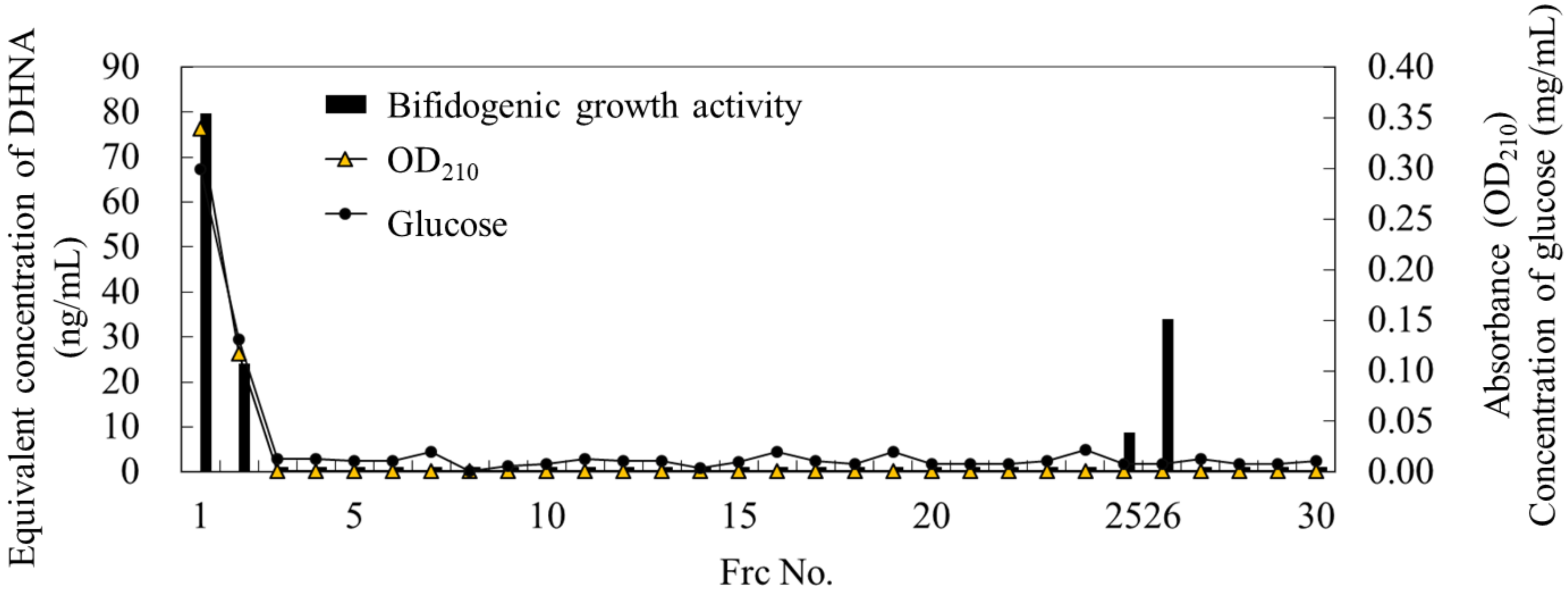

Supplementary Figure 1.

Bifidogenic growth activity, OD210 (as an indicator of amino acid content), and glucose concentration measured for each HPLC fraction. Bifidogenic growth activity is reported as the equivalent concentration of DHNA.

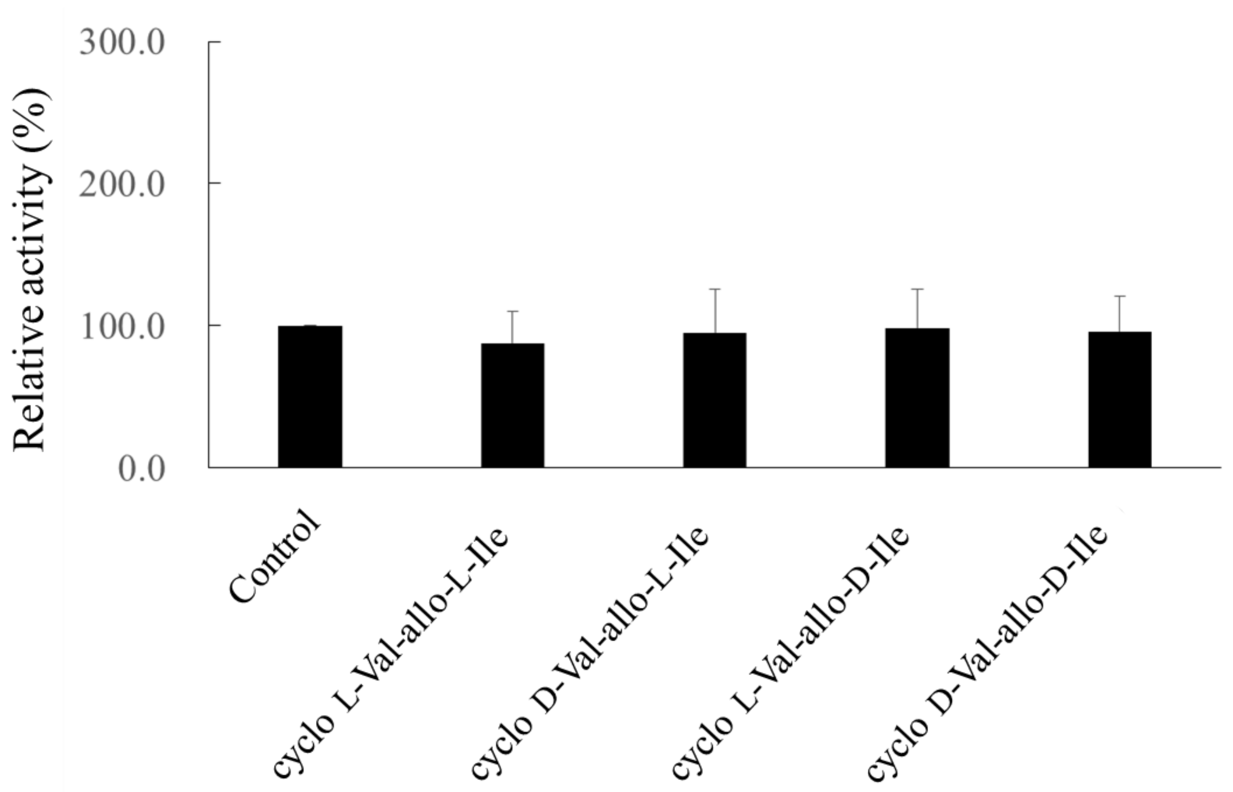

Supplementary Figure 2.
Growth of *B. adolescentis* in response to cyclic dipeptides containing allo-isomers of Ile. Each of the four bifidogenic growth factors containing L-allo-Ile and D-allo-Ile was added, using ethanol as a control, and colony formation was compared. Colony formation of the control was set at 100%. Error bars show the standard error of the mean.

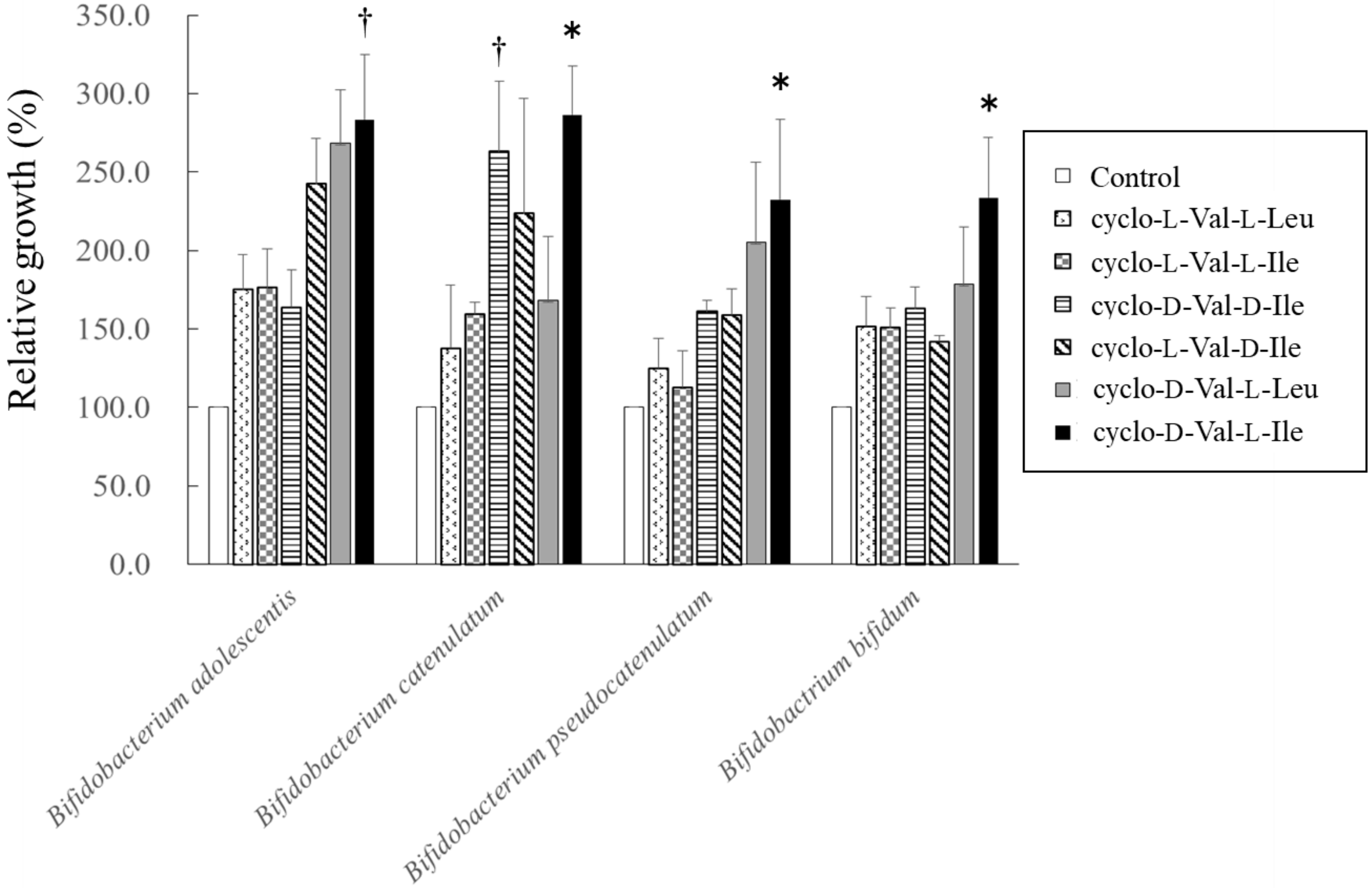

Supplementary Figure 3.

Growth activity of four *Bifidobacterium* species (*B. adolescentis*, *B. catenulatum*, *B. pseudocatenulatum* and *B. bifidum*). Each of the six bifidogenic growth factors was added, using ethanol as a control, and colony formation was compared. Colony formation of the control was set at 100%. Significant differences were determined by Dunnett test (*: P <0.05, †: P <0.1). Error bars show the standard error of the mean.

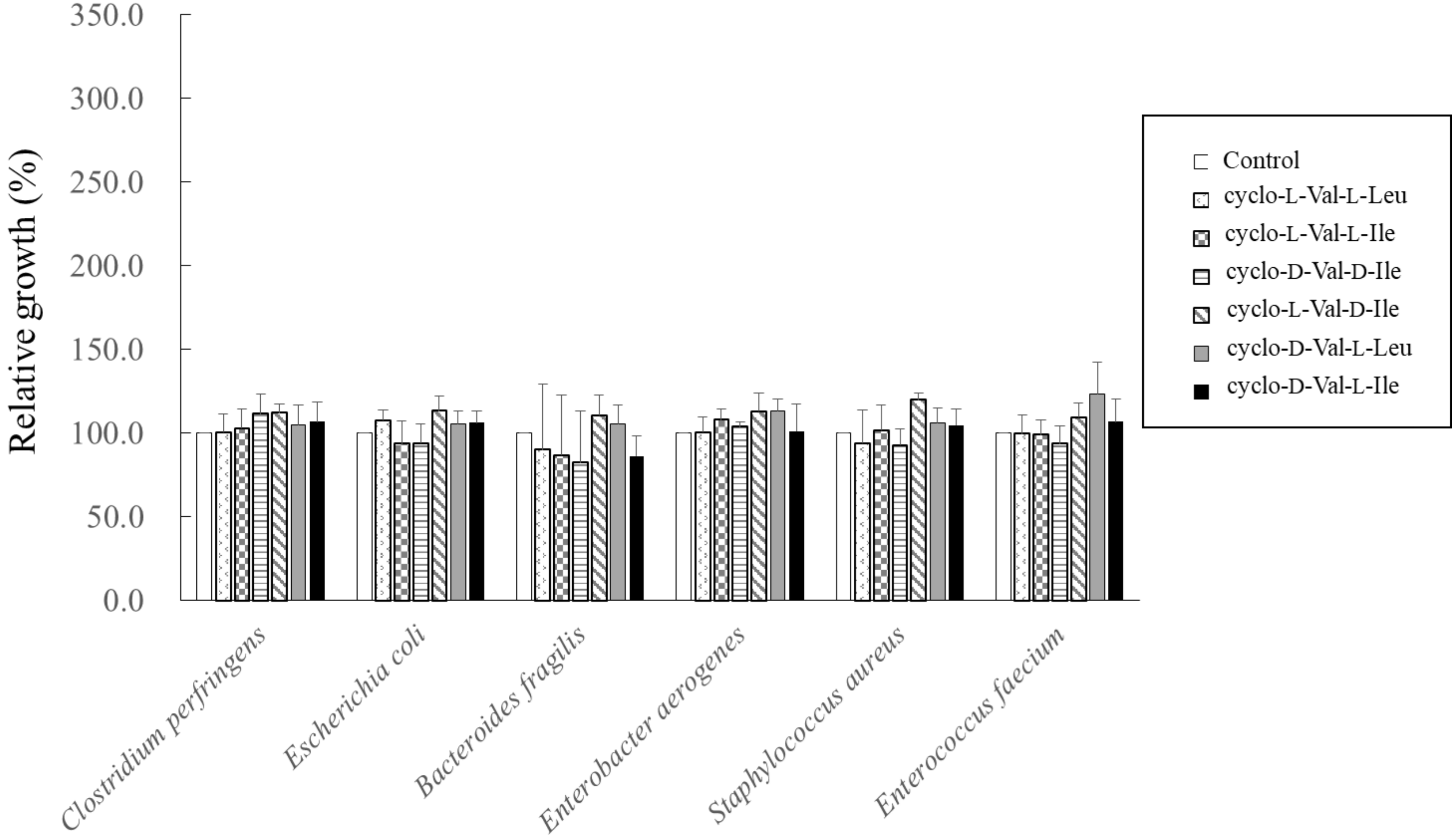

Supplementary Figure 4.

Growth of six harmful gut bacteria (*Clostridium perfringens*, *Escherichia coli*, *Bacteroides fragilis*, *Enterobacter aerogenes*, *Staphylococcus aureus*, and *Enterococcus faecium*) in response to bifidogenic growth factors. Each of the six bifidogenic growth factors was added, using ethanol as a control, and colony formation was compared. Colony formation of the control was set at 100%. Error bars show the standard error of the mean.

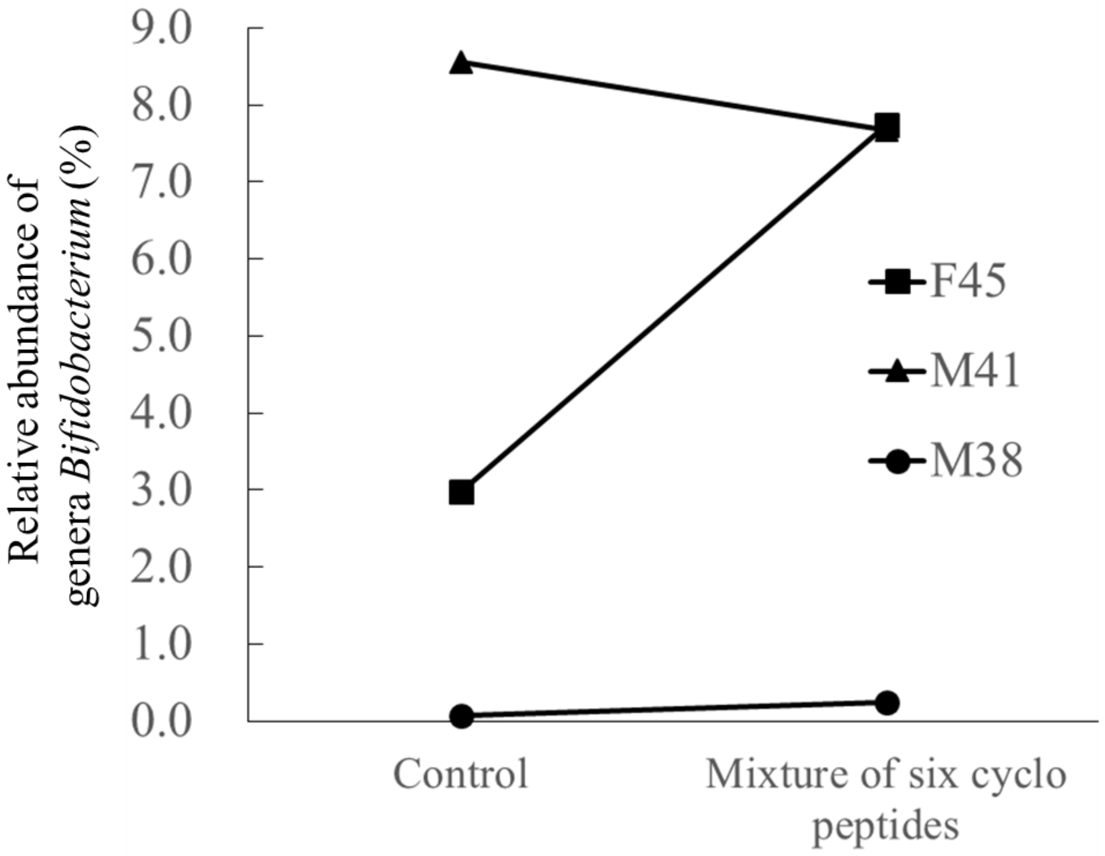

Supplementary Figure 5.
The relative abundance of *Bifidobacterium* in the *in vitro* human colonic microbiota model with a mixture of six cyclo peptides. Fermentation was initiated by inoculating each of three human fecal samples (F45, M41, and M38). A mixture of six bifidogenic growth compounds was added after 6 hours of incubation and no addition was used as the control. After collecting the culture medium after 30 hours of incubation, the microbiota composition was determined by high speed sequencing.
